# Extracellular DNA—A Danger Signal Triggering Immunothrombosis

**DOI:** 10.3389/fimmu.2020.568513

**Published:** 2020-10-07

**Authors:** Chongxu Shi, Luying Yang, Attila Braun, Hans-Joachim Anders

**Affiliations:** ^1^Renal Division, Medizinische Klinik und Poliklinik IV, Klinikum der Universität München, Ludwig-Maximilians University Munich, Munich, Germany; ^2^German Center for Lung Research, Walther-Straub-Institute for Pharmacology and Toxicology, Ludwig-Maximilians University Munich, Munich, Germany

**Keywords:** thrombosis, leukocytes, platelets, vasculitis, mouse model, stroke, sepsis

## Abstract

Clotting and inflammation are effective danger response patterns positively selected by evolution to limit fatal bleeding and pathogen invasion upon traumatic injuries. As a trade-off, thrombotic, and thromboembolic events complicate severe forms of infectious and non-infectious states of acute and chronic inflammation, i.e., immunothrombosis. Factors linked to thrombosis and inflammation include mediators released by platelet granules, complement, and lipid mediators and certain integrins. Extracellular deoxyribonucleic acid (DNA) was a previously unrecognized cellular component in the blood, which elicits profound proinflammatory and prothrombotic effects. Pathogens trigger the release of extracellular DNA together with other pathogen-associated molecular patterns. Dying cells in the inflamed or infected tissue release extracellular DNA together with other danger associated molecular pattern (DAMPs). Neutrophils release DNA by forming neutrophil extracellular traps (NETs) during infection, trauma or other forms of vascular injury. Fluorescence tissue imaging localized extracellular DNA to sites of injury and to intravascular thrombi. Functional studies using deoxyribonuclease (DNase)-deficient mouse strains or recombinant DNase show that extracellular DNA contributes to the process of immunothrombosis. Here, we review rodent models of immunothrombosis and the evolving evidence for extracellular DNA as a driver of immunothrombosis and discuss challenges and prospects for extracellular DNA as a potential therapeutic target.

## Introduction

Evolution positively selected four major danger response programs, i.e., inflammation, clotting, epithelial healing, and mesenchymal healing because they assure survival upon traumatic injury (1). Blood clotting and inflammation are early responses that immediately create barriers. Clotting creates an inside-out barrier for blood loss and clotting and inflammation both create an outside-in barrier for pathogen entry. Balanced clotting can prevent fatal bleeding and balanced inflammation can prevent fatal sepsis. However, trade-offs exist and largely contribute to prevalent disease pathomechanisms in clinical medicine (2). Thrombotic and thromboembolic events are important complications in severe forms of infectious and non-infectious states of acute and chronic inflammation, i.e., immunothrombosis (3). Proinflammatory mediators released from platelets, complement, and lipid mediators link clotting and inflammation as do certain integrins, and neutrophil extracellular traps (NETs).

As a novel entry, extracellular deoxyribonucleic acid (DNA) can elicit profound proinflammatory and pro-thrombotic effects in the extracellular space (4). Pathogens release DNA together with other pathogen-associated molecular patterns (PAMPs). Dying parenchymal cells release extracellular DNA together with other danger- or damage-associated molecular patterns (DAMPs) and neutrophils release DNA by forming neutrophil extracellular traps (NETs) during infection, trauma or other forms of vascular injury (5–7). Extracellular DNA localizes to the sites of injury and experimental studies employing deoxyribonuclease (DNase)-deficient mouse strains or recombinant DNase demonstrate a functional contribution of this extracellular DNA to the development of immunothrombosis.

In this review, we summarize basic knowledge about the process of immunothrombosis, and discuss the role of extracellular DNA as a modulator of thrombosis in the arterial and venous segments of the vasculature. Furthermore, we describe several mouse models to study the process of immunothrombosis in different disease settings.

### Experimental Models of Venous Thrombosis

Venous thrombosis is a common clinical health care problem and causes congestion and pain when affecting the deep veins of the limbs or acute thoracic pain, dyspnea, and shock when affecting the pulmonary arteries. Pulmonary embolism is a severe life-threatening complication of deep vein thrombosis. Venous thrombosis frequently develops in the perianal venous plexus region and usually presents as painful swelling at the site of the blood clot. The most popular rodent model of venous thrombosis is obstruction of the inferior vena cava via a surgical intervention, which generates clots of sufficient size for measuring clot weight and for histopathological characterization of the clot material (8). Here, we introduce several types of venous thrombosis models which are studied in mice (Table 1).

**Table 1:** Animal models of immunothrombosis.

**Model**	**Strengths**	**Weaknesses**	**References**
**Venous thrombosis**			
IVC ligation model (stasis model)	Thrombus size is highly consistent.	It completely blocks blood flow.	(9–15)
IVC stenosis model	Thrombus reduces blood flow, endothelial cell damage.	Thrombus formation is strain-dependent, clamp relevant injury is unclear.	(16–22)
Modified IVC stenosis model	Thrombus reduces blood flow, no endothelial cells damage.	Variable in thrombus incidence and size.	(16–22)
Electrolytic IVC model (EIM)	Thrombus size is highly consistent, with no endothelial cells damage.	Long operation time.	(23–30)
FeCl_3_ IVC model	Produces thrombus within minutes, thrombus size is time-dependent.	Transmural vein wall injury, the thrombus is small, only be used to study early time points.	(31–34)
Recurrent IVC model	Most clinically relevant.	Twice surgeries on the same mouse.	(35)
**Arterial thrombosis**			
Photothrombotic model	Localize the ischemic lesion, minimal variation in infarction, low mortality and invasiveness, highly reproducible.	The translational impact is poor.	(36–47)
Thromboembolic clot models	Any kind of embolus-like material can be used, perfectly matches human embolic stroke.	High variability in infarct size, embolic material not lysisable, high price.	(48–52)
Microsphere/macrosphere model	Infarcts with penumbras, induce ischemic cell death and inflammation. Occlusion can be postponed.	Permanent ischemia, multiple vessels occluded, blood flow redistribution, immediate disruption of the blood-brain barrier and vasogenic edema.	(53–55)
Cholesterol clot model	Cholesterol crystal triggers clots formation, appropriate for thrombolytic agent study, low mortality, low invasiveness, highly reproducible.	Requires a high degree of surgical skill, the high variability of infarct size, localized ischemic region.	(56)
**Thrombotic microangiopathy**			
Acquired TTP model	A simple approach leads to salient features of TTP.	It requires rabbit or mouse antibodies.	(57–61)
Hereditary TTP model	Spontaneous thrombocytopenia	High mortality.	(61)
HIT/T model	Severe thrombocytopenia, allowing pre-clinical studies.	Needs high doses of heparin, Four factors (Heparin, hPF4, FcγRIIA, and anti-heparin/hPF4 antibodies) are present simultaneously.	(62–65)
**Disseminated intravascular coagulation (DIC)**			
Sepsis-related DIC model	Inducible DIC with multiple organ failure, suitable for candidate drugs testing.	Mice are relatively resistant to endotoxin. Needs more than bolus injection.	(66–74)
CLP-related DIC model	Inducible DIC with multiple organ failure. Technically easy, reproducible and similar to human disease.	High mortality and variability.	(75–79)

*IVC, inferior vena cava; TTP, thrombotic thrombocytopenic purpura; HIT/T, heparin-induced thrombocytopenia/thrombosis; CLP, cecal ligation and puncture*.

#### Inferior Vena Cava Stasis Occlusion Model

The stasis occlusion variant is a model of permanent inferior vena cava (IVC) ligation, mimicking the clinical condition of complete vascular occlusion. Technically, the IVC and all collateral side branches distal to the left renal vein are ligated. Thrombus formation in this model involves venous stasis and local release of tissue factor (TF) with augmented coagulation inside the IVC (9, 80). The advantages of this model are its low mortality, high frequency of thrombus generation, and highly consistent thrombus sizes (10, 11). Ultrasound can sequentially monitor thrombus progression and to select an optimal time point for harvesting the thrombus. This model has proven valuable to study the interactions between the venous wall and thrombus progression from acute (first 2–3 days) to chronic inflammation as well as to study the subsequent remodeling of the venous wall (12, 13). As a relevant discrepancy to most venous thromboses in human, blood flow does not establish. Peternel et al. used the stasis occlusion model in rats and found it well-suited for evaluating antithrombotic therapies (14).

#### Inferior Vena Cava Stenosis Model

Partially reducing rather than completely blocking venous blood flow is more similar to the process of venous thrombus in humans. Technically, this implies only partial ligation of side branches of the IVC and using a wire as a spacer during IVC ligation that, once removed, maintains a small lumen with a residual flow avoiding endothelial cell damage (15–21). These subtle modifications mimic a residual flow that is typical for human venous thrombosis and critical for its pathophysiology. As a disadvantage, the thrombus formed in the IVC is generally smaller and the size is more variable (22). This model allows us to better study early thrombotic events (16).

#### Electrolytic Inferior Vena Cava Thrombosis Model

Cooley et al. first described thrombus induction by electrical injury to the common femoral vein of mice (23, 24). Diaz et al. modified the protocol by applying a constant current to a copper wire inserted into the IVC. The electrical current induces free radicals inside the wire, which subsequently activate endothelial cells with minor cell damage (25–27). A thrombus develops quickly in the direction of the blood flow and thrombus sizes are highly consistent. This venous thrombosis model is used to study pro-thrombotic, anti-thrombotic, and thrombolytic therapies (28–30). Moreover, this model can mimic the early and late stages of venous thrombosis. Disadvantages include long procedure time and potential venous wall injury.

#### Ferric Chloride (FeCl_3_) Inferior Vena Cava Thrombosis Model

Local application of FeCl_3_ solution causes oxidative damage to the surface of the exposed venous wall followed by thrombus formation (31, 32). To achieve this, a small piece of filter paper soaked with a defined concentration of FeCl_3_ solution is applied to the IVC for 3 min (33). As toxin exposure allows only a short observation period, thrombus size is usually small with little thrombus material for evaluation. Gustafsson et al. combined FeCl_3_-induced vessel injury with IVC stenosis in rats to obtain a larger thrombus size (34).

#### Recurrent Inferior Vena Cava Thrombosis Model

Patients with deep vein thrombosis face a high risk of post-thrombotic syndrome and 30% experience recurrent thrombosis with 45% occurring in the ipsilateral leg within the following 10 years. Attempts to model recurrent thrombosis employ first the electrolytic method and 21 days later a secondary thrombus is induced using either a second electric insult or a ligature-based method. At the time of the second intervention, the primary thrombus has been incorporated into the venous wall, and the lumen has recovered. This clinically more relevant model has proven valuable to study the biology of recurrent deep vein thrombosis (35).

### Experimental Models of Arterial Thrombosis

Arterial thrombosis is followed by territorial ischemia and infarcts during spontaneous rupture of atherosclerotic plaques, or in patients with an anti-phospholipid syndrome or with trauma. Arterial thrombosis is the central pathologic mechanism contributing to myocardial infarction and ischemic stroke (81). It is a major health concern in terms of cardiovascular morbidity and mortality and has become an attractive drug target for the treatment of these diseases. A variety of reproducible animal models have been developed to investigate the pathomechanisms of arterial thrombosis (Table 1).

#### Photothrombosis Model of Cerebral Stroke

The photothrombotic model uses a photosensitive dye (e.g., Rose Bengal) that after injection responds to illumination across the intact or thinned skull with laser light of a specific wavelength (36, 37). Light exposure induces the formation of oxygen and superoxide radicals damaging surrounding endothelial cell membranes. Endothelial damage promotes local activation and aggregation of platelets. As a consequence, platelet-rich thrombi occluding cerebral microvessels, and causing cortical ischemic infarcts. The photothrombotic stroke model involves the neuroplasticity of perilesional and contralesional brain tissues (38, 39). Modifications of the classical photothrombotic stroke model mimic also a perilesional penumbra. A ring filter model produces a central area of brain injury surrounded by tissue without thrombosis (40), but whether this model sufficiently reflects the penumbra in a human disease context is still under discussion (41). Other modifications include the targeting of individual brain arterioles or implantable optical fibers to produce small subcortical infarcts (42, 43), which surround areas of hypoperfusion with characteristics resembling an inverted penumbra (40). The photothrombotic stroke model allows real-time analysis of many parameters in freely moving rats and mice with acute stroke without the need for anesthesia (44, 45). The activating light can be placed into the specific cortical region of the desired brain area. Using this *in vivo* model, highly reproducible infarct size and low mortality are suitable to study repair mechanisms of the brain and related long-term functional outcomes (46, 47). Since microvascular clots are unusually platelet-rich recombinant tissue plasminogen activator (t-PA) can resolve such clots to a limited extent (42).

#### Thromboembolic Stroke Model

This model is frequently referred to study human thromboembolic stroke, injecting thrombus-like materials into the cerebral vessels, and the internal carotid artery (48, 49). Depending on the size and amount of the injected material, this model is characterized by leading to one or multiple arterial occlusions followed by ischemic infarcts in the respective territory. Compared to models of middle cerebral artery occlusion (MCAO), cerebral thromboembolism models-induced brain infarcts are surrounded by a well-defined penumbra but infarct sizes are more variable. This model is suitable to study the pathomechanisms of arterial immunothrombosis and the effects of thrombolytic drugs in this process (50, 51). However, the emitting source of the embolus is still not part of this model (52).

#### Microsphere/Macrosphere Embolic Stroke Model

Embolic stroke can also be induced by injection of synthetic large-sized macrospheres (diameter between 300 and 400 μm) or small-sized microspheres (diameter between 20 and 50 μm) into the cerebral artery. Many different materials, such as silicone, collagen, and titanium dioxide have been used to induce embolic stroke *in vivo* (53). This model has been characterized by permanent ischemia as the fibrinolytic system cannot dissolve such spheres. Microspheres cause multifocal and heterogeneous small infarcts due to microembolization into multiple arteries (54). Unlike thrombus formation, microspheres block blood vessels suddenly, leading to a rapidly developing edema and redistribution of blood flow (55). Although the macrosphere model induces similar infarct development to the ligation models, it does not allow to study the effect of thrombolytic drugs.

#### Cholesterol Embolism Model

We recently developed a model of cholesterol embolism by injecting cholesterol crystals into the left renal artery of mice (56). According to the size and number of cholesterol crystals, intra-arterial injection leads to multiple microvascular thrombotic occlusions followed by ischemic territorial infarcts. Interestingly, these occlusions are sensitive to thrombolytic therapy preventing tissue infarction and kidney failure. However, it does not appear to be the crystals themselves but rather the blood clots surrounding the crystals that cause vascular obstruction, tissue ischemia, and organ failure (56). As a disadvantage, infarct size is highly variable in this model but the degree of organ failure tightly correlates with the injected crystal dose.

### Experimental Models of Thrombotic Microangiopathy

Thrombotic microangiopathies (TMAs) are heterogeneous disorders characterized by thrombocytopenia, microangiopathic hemolytic anemia, renal failure, and neurological symptoms (82). Complex histopathological features have been detected in TMAs, including thrombosis in arterioles and capillaries with abnormalities in the endothelium and vessel wall [Table 1; (83)]. TMAs can result from having numerous different pathophysiological mechanisms resulting in a spectrum of distinct but frequently overlapping clinical presentations, as discussed in detail elsewhere (84). An important element is genetic and acquired complement system alterations that either alone or in combination with other triggers cause TMA. Such triggers of uncontrolled complement activation at the endothelial interface include infections, bacterial toxins, certain drugs, and malignancies. Placental as well as maternal factors can trigger TMA during pregnancy that can present with different clinical features referred to by a traditional nomenclature, i.e., (pre-) eclampsia or hemolysis-elevated liver enzymes and low platelet count (HELLP) syndrome. Another entity relates to the von Willebrand factor (vWF) cleaving protease disintegrin and metalloproteinase with a thrombospondin type 1 motif, member 13 (ADAMTS13)-induced damages. Given this complexity of disease pathomechanisms, animal models of TMA can mimic only selective scenarios of the broad clinical spectrum of TMA. Some are presented here.

#### Thrombotic Thrombocytopenic Purpura (TTP) Models

TTP develops from absence or inactivation of the ADAMTS13, leading to the accumulation of vWF multimers and the formation of microvascular thrombi with ischemic end-organ damage (57, 82). Two important mouse models have been developed to study the ADAMTS13 function *in vivo*. The TTP-ADAMTS13 proteolytic activity inhibition model is based on the administration of human anti-ADAMTS13 recombinant single-chain variable region antibody fragments (scFv's), which inhibits the enzymatic activity of ADAMTS13 in mice (58, 59). This *in vivo* treatment leads to persisted ADAMTS13 deficiency for over 2 weeks and the formation of microvascular thrombi (58, 60). Administration of Shiga toxin-2 to these mice results in lethal TMA affecting the brain, heart, and kidney (61). In another mouse model, ADAMTS13-deficient mice are challenged with a second hit to develop TTP, e.g., the infusion of Shiga toxin causes a syndrome closely resembling human TTP with widespread vWF-rich and fibrin-poor hyaline thrombi in the microvasculature of multiple organs (62).

#### Heparin-Induced Thrombocytopenia (HIT) and Thrombosis

Heparin can trigger an immune-mediated thrombocytopenic disorder characterized by venous and arterial thrombus formation via antibodies against complexes of human platelet factor 4 (PF4) and heparin (63, 64). Heparin-induced thrombocytopenia in mice requires transgenic expression of human PF4 and a lack of the genetic equivalent of human Fc gamma receptor IIA (FcRIIA). As a third requirement, mice are injected with anti-heparin-PF4 immunoglobulin (IgG) and heparin (64, 65, 85). Although this combination of causal factors is not identical to the clinical scenario in patients, the mouse model is suitable to study HIT. Also, in some cases, lethal TTP with disseminated arterial and venous thrombi have been described in mouse models of HIT.

#### Disseminated Intravascular Coagulation (DIC) Model

Thrombocytopenia is frequently observed in septic patients who have a systemic activation of immunothrombotic mechanisms (66, 86, 87). Several important models have been developed to study the pathology of DIC in mice. In the endotoxemia model, injection of lipopolysaccharide (LPS), zymosan or *E. coli* bacteria in mice initiates an overwhelming activation of innate immunity and procoagulant pathways that can lead to DIC with multiple organ dysfunction (67–70). Pathophysiological characteristics of this treatment are reduced platelet count, prolonged bleeding time, decreased plasma fibrinogen levels, and increased plasma D-dimer levels (71–73). This model is often used for the testing of drug candidates (74, 75).

#### Caecal Ligation and Puncture Model

This represents the gold standard for research on polymicrobial sepsis (76, 77). It consists of DIC-like microvascular thrombosis and multiple organ failure representing an irreversible stage of sepsis (78, 79).

### Cellular Components and Molecular Mechanisms of Immunothrombosis

#### Platelets

**Figure 1:**
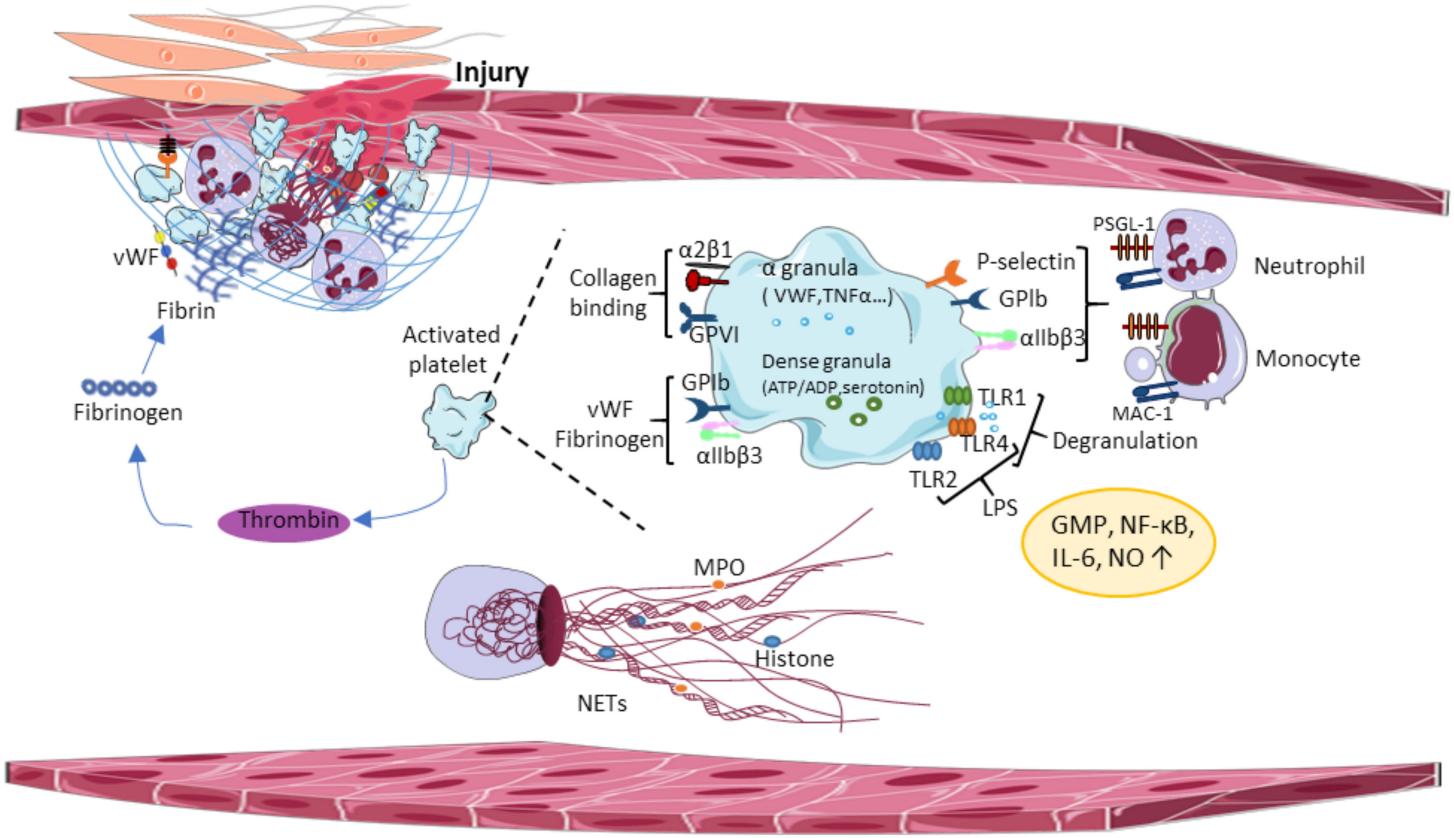
Central paradigms of immunothrombosis. Damaged vessel walls and injured endothelial cells release tissue factor (TF) and extracellular matrix molecules, inducing functional crosstalk between platelets and leading to platelet aggregation. Activated platelets promote thrombin formation thereby enhancing platelet degranulation and fibrin formation. Activated platelets release proinflammatory cytokines from α-granules, which promotes platelet-neutrophil interaction and triggers the release of the NETs. vWF, von Willebrand Factor; GPIb, glycoprotein Ib; GPVI, glycoprotein VI; TLR, toll-like receptor; LPS, lipopolysaccharides; NO, nitric oxide; GMP, guanosine monophosphate; NF-κβ, nuclear factor-κ beta; IL-6, interleukin 6; MPO, myeloperoxidase; NETs, neutrophil extracellular traps; MAC-1, macrophage antigen 1.

Studies using mouse models of sepsis revealed the accumulation of platelets in the microvasculature. Indeed, LPS injection resulted in thrombocytopenia in mice, the accumulation of platelets was found in the lung and liver. Several Toll-like receptors (TLRs) were identified in human and mouse platelets to bind a major component of the wall of gram-negative microorganisms (LPS), transmitting signals between platelets and the innate immune system, thereby inducing inflammatory responses. TLR-2 and−4 on human and mouse platelets bind LPS and increase nitric oxide and cyclic guanosine monophosphate (GMP) levels, and activate protein kinase G (88). TLR4 activates the nuclear factor-κB (NF-κB) and the mitogen-activated protein (MAP) kinases increasing interleukin6 (IL-6), cyclooxygenase (COX-2), and prostaglandin E2 (PGE2) production (89). Platelet TLR-1- and−4 are involved in the development of microvascular thrombosis and sepsis-induced intravascular coagulation by triggering platelet degranulation, which releases proinflammatory cytokines from alpha (α)-granules and promotes platelet-neutrophil interaction (Figure 1). Several other isoforms of TLRs have been studied in human and mouse platelets, connecting TLR signaling to pathogenesis of virus-induced thrombocytopenia, and intravascular coagulation. Platelet glycoprotein (GP) Ib and αIIbβ3 integrins are involved in this process, together with an extracellular matrix bridge formed by vWF and fibrinogen. It has been shown that collagen-mediated-activation of GPVI signaling in platelets plays an important role in platelet adhesion onto the inflamed endothelium (90). Altogether, these results suggest that platelets can distinguish between cellular immunity and hemostasis using a combination of different platelet TLRs and, depending on the ligand binding of the pathogens, platelet TLRs can transduce effector signals to immune cells.

#### Endothelial Injury, TF, and Thrombus Formation

In the inflamed vessel wall, endothelial cells start to expose TF and extracellular matrix molecules at the luminal surface, which induces functional crosstalk between platelets, immune cells and activates the coagulation cascade. Platelets and neutrophils are the first blood cells adhering to the luminal endothelial surface of the inflamed vessel wall. Platelet GPIb binds vWF, GPVI binds collagen, laminin, and fibrin (91, 92). Besides these interactions, α2β1 integrin and GPV bind collagen, and α6β1 interacts with laminin during thrombus formation (93, 94). Thrombus growth involves additional platelet recruitment, thereby accelerating the coagulation cascade and the immune response, which stabilizes the growing thrombus on the endothelium surface (Figure 1). Thrombin generation amplifies platelet granule secretion priming the innate immune system. Granule-resident factors released by platelets have diverse effects on the innate immune system, including monocyte cell differentiation (95), neutrophil cell migration (96), phagocytosis, and cytokine responses. For example, platelet granules contain second wave mediators [adenosine triphosphate (ATP) and serotonin], plasma factors, TFs, fibrinogen, and (pro)-inflammatory cytokines, and chemokines. Platelet serotonin released from delta (δ)-granules could significantly increase neutrophil accumulation and extravasation during inflammation (97). Platelet inflammatory cytokines interleukin-1 (IL-1), regulated upon Activation, Normal T Cell Expressed and Presumably Secreted (RANTES), platelet-derived growth factor (PDGF), transforming growth factor-β (TGFβ), and epidermal growth factor (EGF) enhance local inflammatory responses and supported by platelet chemokines, such as chemokine (C-C motif) ligand 5 (CCL5), chemokine (C-X-C motif) ligand 4 (CXCL4), and 7 (CXCL7) that activate monocytes and neutrophils. Interestingly, monocytes express TF in certain pathological conditions (98, 99), connecting the innate immune system to the coagulation cascade. In addition, microparticles released by monocytes bind the platelet surface to accumulate TFs and to promote coagulation (100).

#### Platelet-Immune Cell Interactions

Platelet attachment to the inflamed vessel walls is supported by the interaction between platelets and extracellular matrix components, thereby promoting the interaction of platelets with immune cells and endothelial cells. Indeed, the interaction of surface receptors of activated immune cells and platelets strongly influences innate immune responses. It has been shown that liver-resident macrophages (Kupffer cells) can interact with platelets via platelet GPIb receptor and the exposed vWF on the Kupffer cell surface, e.g., during bacterial infection-induced immunothrombosis (101). Such platelet-immune cell conjugates correlate to the severity of sepsis (102). Although the detected lifetime of this conjugates in the peripheral blood is very short, this interaction activates integrins and induces granule secretion. Platelet-neutrophil adhesion to the endothelium involves the interaction between neutrophil P-selectin glycoprotein ligand-1 (PSGL-1) and αMβ2 integrin to platelet P-selectin and GPIb, respectively. Neutrophil macrophage antigen 1 (Mac-1) also binds platelet GPIb and αIIbβ3 integrin in the presence of fibrinogen, inducing exposure of proinflammatory chemokines CXCL4 and CCL5 (103). P-selectin and PSGL-1 interactions also contribute to the formation of platelet-monocyte conjugates resulting in monocyte activation. Also, monocyte-resident Mac-1 receptor and platelet adhesion receptors GPIb, junction adhesion molecule 3 (JAM3), or αIIbβ3 integrin form transient interactions (104).

#### Platelets and NET Formation

Electron micrographs showed adhesion and aggregation of activated platelet within a fibrous meshwork of NETs (105). In addition, platelet aggregation accurred in a time-dependent manner, and DNase treatment simultaneously cleared NETs and platelets (105). The release of NET into the circulation is followed by platelet adhesion and aggregation, which together with histones released from NETs promote thrombus formation and growth (4). The NET release was observed also in non-infectious inflammatory conditions, such as venous-, microvascular-, and cancer-related thrombosis, acute lung injury, endothelial damage with trauma, autoimmune diseases, preeclampsia, and systemic lupus erythematosus (106). Interestingly, the structure of thrombi in the presence of NETs is more rigid and less permeable. In a mouse model of sepsis, TLR2, and TLR4 on the surface of platelets in liver sinusoids and lung capillaries contribute to platelet-neutrophil interaction and NET formation (107). In addition, synchronized activation of surface integrins and chemokine receptors induce NET formation (108). Thromboxane A2 (TxA2)-released from activated platelets can also amplify NET formation and this process is inhibited by aspirin (109).

#### Coagulation

The blood coagulation cascade operates in three steps: (i) formation of prothrombin activator, (ii) conversion of prothrombin to thrombin, and (iii) conversion of fibrinogen to fibrin (Figure 1). The first step involves the intrinsic coagulation pathways. The intrinsic pathway is activated by exposure of endothelial collagen and the extrinsic pathway is activated through TF released by injured endothelial cells. The following two steps encase platelet aggregates and red blood cells into a fibrin network and attach it to the damaged endothelium. At sites of the damaged vessel wall, platelet activation and degranulation convert inactive IL-1 to the active form by thrombin cleavage, thereby connecting the coagulation system to immunothrombosis. NET release influences the coagulation cascade by activating coagulation factor XII (FXII), inactivating anticoagulant tissue factor pathway inhibitor (TFPI), and by providing an active surface for platelet adhesion and aggregation. All of these mechanisms lead to the inhibition of fibrinolytic activity, thereby promoting thrombus formation and growth.

## Pro-Thrombotic Danger-Associated Molecular Patterns (DAMPs): The Role of Extracellular DNA in Immunothrombosis

Among the mediators released from injured cells, extracellular DNA acts as pro-thrombotic DAMP (110–112). Released chromatin forms similar functional structures as the fibrin network to trap red blood cells, platelets, and coagulation factors including TF and fibrin (113). Here, we discuss some experimental pieces of evidence derived from studies on venous, arterial and microvascular thrombosis, and ischemic stroke (Table 2).

**Table 2:** Experimental evidence for the role of extracellular DNA in immunothrombosis.

**Model**	**Type of evidence**	**References**
Venous thrombosis	IVC model, ecDNA were present in thrombosis, DNase degrades ecDNA, breaks down NETs, reduces thrombus size.	(16, 114, 115)
	Acute limb IRI model, DNase I reduced DNA traps, inflammation, Thrombin-Anti-Thrombin-III expression, and enhanced post-ischemic hind limb perfusion.	(116)
	*Ex vivo*, DNA-histones complexes improved stability and rigidity of thrombus, and DNase promotes clot lysis.	(117)
Arterial thrombosis	Murine models of atherosclerosis, DNase I reduced atherosclerosis burden.	(118)
	Ischemic stroke model, circulating nucleosomes and DNA was increased after ischemic stroke. DNase I reduced infarct size and improved stroke outcome.	(119)
	Cholesterol clot model, ecDNA were presented in crystal clots, DNase prevented clots formation, reduced organ infarction.	(56)
	Thrombi collected from stroke patients, neutrophils were abundant in all thrombi, and NETs contributed to the composition of all thrombi especially in their outer layers.	(120, 121)
Thrombotic microangiopathies syndromes	HIT/T model, thrombi including neutrophils, extracellular DNA. While neutrophil depletion abolishes thrombus formation, DNase treatment limited venous thrombus size.	(122, 123)
	Sepsis-induced DIC in the murine model, ecDNA were presented in thrombus, the blood vessel of lung occluded in DNase deficient mice, DNase treatment prevented NETs clot. Time-dependent increase of cfDNA, later administration of DNase reduced cfDNA, inflammation, and suppressed organ damage.	(124, 125)
	In a murine CLP model, later administration of DNase 4 or 6 h after CLP resulted in reduced cell-free DNA, inflammation, prevented organ damage, and improved survival.	(126)
	In acute TMA patients, levels of DNase activity of plasma showed a significant reduction in compared with healthy controls, plasma-mediated degradation of NETs is reduced in patients with acute TMA.	(127)

*IVC, inferior vena cava; ecDNA, extracellular DNA; cfDNA, cell-free DNA; NET, neutrophil extracellular trap; HIT/T, heparin-induced thrombocytopenia/thrombosis; CLP, cecal ligation and puncture*.

### Contribution of Extracellular DNA to Venous Thrombosis

Several animal models established the role of extracellular DNA in venous thrombosis (Table 2). Ligation of the IVC in mice can increase plasma levels of extracellular DNA during several days (114). Immunofluorescence studies revealed colocalization of extracellular DNA with histones and vWF in the thrombus. DNase I administration protected mice from thrombosis at 6 h and 48 h in this model, indicating that the extracellular DNA itself is a critical component of fibrin-rich thrombi. Several experimental studies confirmed the presence of extracellular DNA in venous thrombi induced by the restriction of blood flow (16, 114, 115). In a mouse model of acute limb ischemia-reperfusion injury, DNase I treatment significantly reduced the presence of extracellular DNA traps, immune cell infiltration, thrombin-anti-thrombin-III generation, and enhanced post-ischemic hind limb perfusion. Interestingly, neutrophil depletion resulted only in a small reduction in DNA traps without inducing any skeletal muscle injury, or hind limb perfusion (116). Indeed, *ex vivo* experiments showed that DNA-histone complexes stabilized the fibrin network resulting in a higher rigidity of an artificial thrombus that was resistant to t-PA. In contrast, adding DNase I promoted clot lysis in combination with t-PA (117). Thus, evolving data in a set of different models of venous thrombosis consistently demonstrated a role of extracellular DNA in venous thrombosis.

### Contribution of Extracellular DNA to Arterial Thrombosis

Numerous studies on animal models suggested the role of extracellular DNA in arterial thrombosis. In murine models of atherosclerosis, DNase I treatment resulted in a reduced burden of atherosclerosis (118). Recently, we showed that in a murine model of cholesterol embolism, extracellular DNA can be a non-redundant component of crystal clots forming within a few hours upon embolization and vascular occlusion. Similar to the platelet purinergic receptor P2Y12 antagonism with clopidogrel, DNase I treatment significantly reduced the numbers of obstructed arteries, decreased ischemic-related organ failure, and tissue infarction (56). In addition, preincubation of washed platelets with DNAse I inhibited platelet activation, P-selectin exposure, and aggregation response to a collagen-related peptide (CRP) and thrombin. Furthermore, treatment with DNAse I inhibits ATP release and the formation of a fibrin network.

### Contribution of Extracellular DNA in Ischemic Stroke

In stroke patients, extracellular DNA components have been also observed in ischemic brain tissues, possibly contributing to stroke development. In support of this, histological analysis of thrombi collected from stroke patients revealed that a large number of nucleated leukocytes presented in all thrombosis specimens, neutrophils were abundant in all observed thrombi, and NETs were found in all thrombi, in particular in their outer layers (119, 120). In a murine model of transient middle cerebral artery occlusion (tMCAO), increased levels of circulating nucleosomes and DNA were found after ischemic stroke. Under hypoxic conditions, an increased level of extracellular chromatin was detected. Moreover, targeting extracellular chromatin components with DNase I improved stroke outcome (121). Strikingly, adding DNase I to t-PA significantly accelerated the *ex vivo* lysis of thrombi compared to t-PA alone (119, 120).

### Contribution of Extracellular DNA to Thrombus Formation in the Microvasculature

Recent experimental evidence suggests that extracellular DNA plays an important role in DIC-related organ dysfunction, probably caused by elevated levels of circulating thrombin, high platelet aggregation, vascular leakage, the release of proinflammatory cytokines, and NET formation (128). In sepsis-induced DIC, large numbers of NETs are accumulated mainly in the microvasculature of the lung and liver (124). Studies using DNase-deficient mice reported that the formation of NET clots associates with TMA and DIC, including schistocytes, hemolytic anemia, and organ failure due to vascular occlusions. Similar observations have been detected in patients with severe bacterial infections (124). Studies using multicolor confocal intravital microscopy studies showed the presence of aggregated platelets and fibrin clots together with extracellular DNA in septic tissues (125). NETosis is regulated by the citrullinating enzyme peptidyl arginine deiminase 4 (PAD4) which induces decondensation of the chromatin through arginine modification of histones. Accordingly, in mouse models of sepsis, deficiency of PAD4, or DNase I treatment significantly inhibited systemic intravascular thrombin activity, reduced platelet aggregation, and improved microvascular perfusion (125). Patients with acute TMA show lower plasma levels of DNase I when compared with healthy controls (127). In a murine caecal ligation and puncture model, a time-dependent increase in cell-free DNA was accompanied by systemic inflammation (126). Interestingly, early administration of DNase I at 2 h after caecal ligation and puncture resulted in a drop in circulating cell-free DNA levels, increased inflammation, and organ damage in the lungs and kidneys. In contrast, later administration of DNase I, 4 or 6 h after caecal ligation and puncture, resulted in less cell-free DNA and inflammation, preventing organ damage and improving survival (126).

In a mouse model of HIT, thrombi are composed of neutrophils, extracellular DNA, citrullinated histone H3, and platelets. Interestingly, neutrophil depletion or Pad4*-*deficiency abrogates thrombus formation and DNase I treatment reduced the size of venous thrombi (122, 123).

As the studies on animal models supported a therapeutic potential of recombinant DNase I against thrombus formation in different types of vessels, this concept deserves further investigation at the clinical level. Recently, clinical studies suggested that endogenous DNase I activity could represent a therapeutic biomarker during acute myocardial infarction (129). Accordingly, coronary NET burden and endogenous DNase activity are shown as predictors of myocardial infarct size and stenosis resolution (130). Indeed, recombinant DNase I can accelerate t-PA-mediated lysis of human coronary and cerebral thrombi *ex vivo* (119, 120). Patients with acute microvascular thrombosis displayed reduced DNase I activity (127). Timely and efficient removal of extracellular DNA is required to prevent excessive thrombus formation. The restoration of plasma DNase I activity possibly represents a new therapy for thrombotic complications.

### Cellular Sources of Extracellular DNA in Immunothrombosis

Extracellular DNA could be released by activated immune cells such as neutrophils and monocytes, by apoptotic platelets or by the damaged vasculature (131–134). Therefore, it is difficult to identify precisely the sources of extracellular DNA that contribute to thrombus formation *in vivo* context. Neutrophils are considered as a major source of extracellular DNA when they release their chromatin as NETs (105, 135). As indicated above, NETs are critical for the development of sepsis-induced intravascular coagulation regardless of the inciting bacterial stimulus (gram-negative, gram-positive, or bacterial products). Indeed, many clinical and experimental studies use extracellular DNA as a marker for NETs in the circulation. NETs and extracellular DNA are present in patients with HIT. In patients with myocardial infarction, blood samples contain DNA, nucleosomes, myeloperoxidase, and neutrophil elastase, and their plasma levels correlated with the burden of NETs, detected within coronary thrombi, as well as with the infarct size (130). In ischemic stroke, thrombi in cerebral arteries stain positive for neutrophils, extracellular DNA, and neutrophil elastase, suggesting NET formation (119).

Extracellular traps are also released from monocytes, referred to as METs. METs have a similar web-like structure comprising DNA, granular enzymes, and citrullinated histones, and procoagulant activity, similar to NETs (132). Besides neutrophils and monocytes, it has been reported that eosinophils also form extracellular traps (134).

Another source of extracellular DNA can be released by necrotic vascular or parenchymal cells. During thrombosis-induced tissue ischemia, the majority of cells die primarily via a process of necrosis, this process releases nuclear DNA into the extracellular space and bloodstream. Injured cardiomyocytes are probably a major source of extracellular DNA in patients with myocardial infarction (136, 137). Another source is injured endothelial cells at the site of vascular obstruction (56, 138). Finally, activated platelets release DNA from their mitochondria. Although the total amount of mitochondrial DNA per platelet is low, the large numbers of platelets involved in blood clotting also render platelets as a potentially significant source of extracellular DNA (139, 140). Taken together, numerous sources contribute to the pool of extracellular DNA in immunothrombosis.

### Therapeutic Potential of Recombinant DNAse I in Immunothrombosis

**Figure 2:**
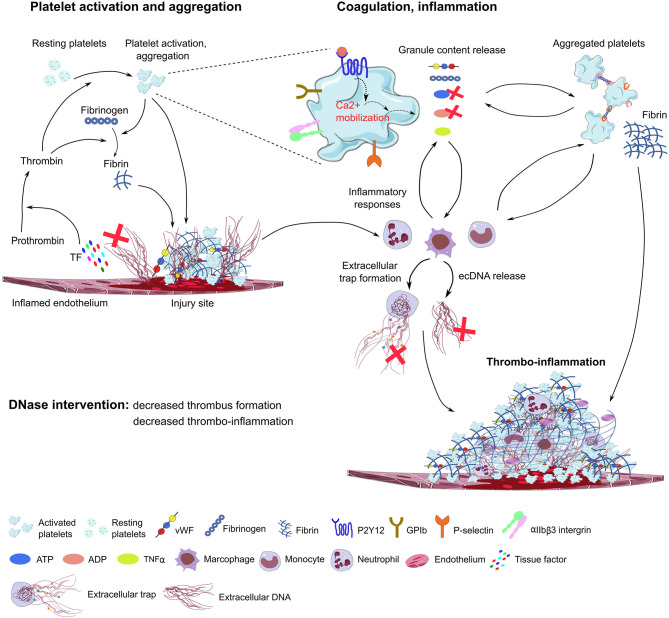
Proposed model of DNase function in immunothrombosis. Damaged endothelial cells release tissue factor (TF) and ecDNA. TF activates the coagulation cascade, converting prothrombin to thrombin, which further activates platelets through PAR receptors. The ecDNA acts as DAMP and directly activates platelets and triggers inflammatory responses. The damaged endothelial layer exposes extracellular matrix proteins (collagen, laminin), and accumulates vWF, fibrinogen and other blood plasma proteins on the endothelial surface, further supporting platelet adhesion and activation through platelet specific glycoprotein receptors (GPIb, GPVI) and integrins (αIIbβ3, α2β1). During degranulation, second wave mediators (ATP, ADP, serotonin), extracellular matrix components (vWF, fibrinogen), and inflammatory cytokines are released by activated platelets, triggering thrombus formation and enhancing immune cell responses and NET formation. Platelet purinergic receptors (P2Y1, P2Y12) are activated by ADP, further promoting platelet aggregation and thrombus growth. P-selectin exposure on the plasma membrane of activated platelets increases procoagulant activity and supports platelet-immune cell interaction and NET formation. In the process of immunothrombosis, DNase could inhibit NETosis by fragmenting DNA within the NETs, thereby dissociating platelet-rich components from the endothelial surface, and inhibiting thrombus growth. DNAse may also inhibit purinergic signals in platelets and immune cells. TF, tissue factor; ec-DNA, extracellular deoxyribonucleic acid; vWF, von Willebrand Factor; ADP, adenosine diphosphate; ATP, adenosine triphosphate; TNFα, tumor necrosis factor alpha (TNFα); GPIb, glycoprotein Ib; GPVI, glycoprotein VI, DNase, deoxyribonuclease; ADP, adenosine diphosphate; ATP, adenosine triphosphate.

In a mouse model of sepsis-induced intravascular coagulation, NET release coincided with increased platelet aggregation, thrombin generation, and fibrin clot formation (125). DNase I treatment reduced NET formation and degraded extracellular DNA, which was associated with inhibited platelet aggregation and microvascular obstructions (Figure 2). In the LPS-induced sepsis mouse model, NET release and fibrin clot formation were inhibited by the combined treatment of DNase I with the thrombin inhibitor argatroban. However, these treatments did not influence bacterial dissemination (141). In line with this, in septic patients, NETs also significantly increased the generation of thrombin and fibrin clot formation, and this effect was reduced by DNase I treatment (142). Of note, DNase I treatment leads to the release of NET components into the bloodstream, which may elicit procoagulant activity and intravascular thrombosis in septic patients. Free extracellular DNA fragments enhance the intrinsic coagulation pathway (143), which is also observed in patients with deep vein thrombosis (144), leading to tissue hypoxia and endothelial damage.

It is known that DNA intercalates with fibrin to form a scaffold that stabilizes clot structure in the bloodstream (4), therefore DNA-fibrin complexes have a fundamental effect on clots lysis. In plasma of septic patients, extracellular DNA significantly delayed t-PA-mediated clot lysis times by forming DNA-plasmin-fibrin ternary complex which results in a densely packed clot structure (145). Elevated levels of extracellular DNA in plasma from septic patients promoted thrombin generation (146). DNA alone or NETs inhibited plasminogen activation and t-PA-induced resolution of plasma clots (147). In a murine model of HIT, PF4 combines with NET-forming complexes that selectively bind HIT-induced antibodies, DNase I treatment limited venous thrombus size (148). Extracellular DNA markedly affects the hemostatic system by activating factor XI (FXI) and factor XII (FXII) (149). Extracellular DNA present in the fibrin clot inhibits the antithrombotic activities of anticoagulants, such as unfractionated heparin and enoxaparin (150). In contrast, RE31 DNA aptamers inhibit thrombin formation, accelerates fibrinolysis *in vitro*, and suppress thrombosis *in vivo* (151, 152).

In cystic fibrosis the lung is frequently affected by recurrent bacterial infections and chronic inflammation causing progressive lung destruction; the development of thick mucus in small bronchioles and peribronchial regions of the lung thereby triggering permanent bacterial infection. Infiltrated neutrophils release granular content to eliminate the pathogens, and also release high concentrations of extracellular DNA, forming NETs in the inflamed bronchioles, which contribute to airway damage, aggravating mucus viscosity, and its mucociliary clearance from the bronchioles. Blood samples from patients with cystic fibrosis showed an increased number of activated platelets (153), which form cell conjugates with monocytes and neutrophils (153, 154). Increased platelet aggregation responses to adenosine diphosphate (ADP) and thrombin receptor-activating peptide (TRAP), and second-wave mediators (TxA2, ATP, serotonin), and α-granule-resident proteins [tumor necrosis factor alpha (TNFα), CD40 ligand (CD40L), leukotriene B4 (LTB4), and interleukins] were also detected (155, 156). Plasma levels of platelet granule-resident proteins are correlated with a decreased lung function of these patients (157, 158). DNase I treatment showed significant improvement in rheological parameters in cystic fibrosis, reducing the thick mucus layer by cleaving the extracellular DNA of NETs. Therefore, patients can release more easily the accumulated mucus up from the inflamed lung tissue.

The literature also describes that following bacteremia, neutrophils recruited to the liver sinusoids enhance the clearance of pathogens from the circulation (107, 159). Similar to the phenomenon observed in septic lung tissues, and in liver sinusoids, neutrophils also release intravascular NETs (69). Blocking NET formation by DNase I reduced the capture of circulating bacteria in the liver, resulting in increased dissemination of bacteria to distant organs.

NET formation was also detected in acute ischemic stroke, located in the outer layer of developing thrombi, and consequently, the increase of extracellular DNA content in the blood plasma correlates with stroke severity. Although thrombolysis with t-PA administration promotes fibrin degradation in the occluded vessel of acute ischemic stroke, t-PA-resistant clot formation has been frequently observed in platelet-rich arterial thrombi. Hence, fibrin accumulation in the growing thrombi is limited at the early phase of acute ischemic stroke (160, 161). Interestingly, the co-administration of DNase I with t-PA accelerated thrombolysis *ex vivo*. However, DNase I treatment alone had no thrombolytic effect *ex vivo*. These results suggest that both fibrin and NET formations can be targeted simultaneously to induce successful thrombolysis and recanalization of the artery in acute ischemic stroke (120). In line with these results, combined treatment of DNase I with t-PA also attenuated infarct size in a murine model of myocardial ischemia-reperfusion injury. Again, DNase I or t-PA treatment alone had no beneficial effects in this mouse model (162). Altogether, these results suggest that DNase I and t-PA treatment together improve both myocardial and cerebral post-ischemic infarction. However, a clinically implemented and safe pharmacological strategy of DNase I treatment is currently established in patients with cystic fibrosis (163) and limited clinical trials investigated the thrombolytic effects of NET degradation in other disease conditions. Altogether, these data suggest that in some cases, DNA-targeted therapies by DNase I may improve thrombolysis and inhibit coagulation. Therefore, further investigation is necessary to establish the role of DNase I treatment in immunothrombosis.

## Conclusions and Future Perspectives

Immunothrombosis is a complex process involving numerous elements of the cascades of coagulation and inflammation. *In vivo* preemptive administration of recombinant DNase I not only cleaves deposits of extracellular DNA but also inhibits ATP release from platelet δ-granules and prevents the formation of fibrin network. Extracellular DNA may directly induce fibrin formation, thereby enhancing thrombus growth. Studies analyzing the role of extracellular DNA in immunothrombosis related to either the use of DNase-deficient mice or the recombinant DNase I. It worth to postulate that DNAse I treatment may limit thrombus formation by inhibiting the function of platelet-derived second wave mediators, such ATP.

Several questions remain unanswered: What is the main source of extracellular DNA during the early phase of blood clotting *in vivo*? How does the extracellular DNA released from infarcted tissues may contribute to the clot formation and the resistance to the fibrinolysis? Is extracellular DNA a suitable therapeutic target in humans beyond the anticoagulants or fibrinolytic drugs? Does recombinant DNase I have a better safety profile compared to the anticoagulants? A better understanding of the role of extracellular DNA in a immunothrombosis context is required to clarify these issues.

## Author Contributions

CS, H-JA, and AB wrote the manuscript and prepared tables. LY and CS prepared the figures. H-JA and AB revised the manuscript. All authors contributed to the article and approved the submitted version.

## Conflict of Interest

The authors declare that the research was conducted in the absence of any commercial or financial relationships that could be construed as a potential conflict of interest.
